# The role of cuticular hydrocarbons in mate recognition in *Drosophila suzukii*

**DOI:** 10.1038/s41598-018-23189-6

**Published:** 2018-03-22

**Authors:** Yannick Snellings, Beatriz Herrera, Bruna Wildemann, Melissa Beelen, Liesbeth Zwarts, Tom Wenseleers, Patrick Callaerts

**Affiliations:** 1 KU Leuven, Department of Human Genetics, Laboratory of Behavioral and Developmental Genetics, Leuven, 3000 Belgium; 2 VIB, Department of Microbiology, Laboratory of Systems Biology, Heverlee, 3001 Belgium; 3 KU Leuven, Department of Biology, Ecology, Evolution and Biodiversity Conservation Section, Leuven, 3000 Belgium

## Abstract

Cuticular hydrocarbons (CHCs) play a central role in the chemical communication of many insects. In *Drosophila suzukii*, an economically important pest insect, very little is known about chemical communication and the possible role of CHCs. In this study, we identified 60 CHCs of *Drosophila suzukii* and studied their changes in function of age (maturation), sex and interactions with the opposite sex. We demonstrate that age (maturation) is the key factor driving changes in the CHC profiles. We then test the effect on courtship behaviour and mating of six CHCs, five of which were positively associated with maturation and one negatively. The results of these experiments demonstrate that four of the major CHC peaks with a chain length of 23 carbons, namely 9-tricosene (9-C23:1), 7-tricosene (7-C23:1), 5-tricosene (5-C23:1) and tricosane (*n*-C23), negatively regulated courtship and mating, even though all these compounds were characteristic for sexually mature flies. We then go on to show that this effect on courtship and mating is likely due to the disruption of the natural ratios in which these hydrocarbons occur in *Drosophila suzukii*. Overall, these results provide key insights into the cuticular hydrocarbon signals that play a role in *D*. *suzukii* mate recognition.

## Introduction

Cuticular hydrocarbons (CHCs) covering the insect cuticle not only protect against desiccation but also play major roles in chemical communication, including in aggregation, species, nest or mate recognition and the signaling of reproductive status^1–5^. CHCs are biosynthesized in the oenocytes, which are secretory cells that are situated in the abdominal epidermis^6–9^, and are typically non- or semi-volatile long-chain hydrocarbons with one or more double bonds or methyl branches. Consequently, when they serve communicative functions they tend to be used primarily as contact or short-range pheromones^1,4,10^.

In Diptera, CHCs have diverse functions, including aggregation and mate and species recognition^11^. In *Drosophila melanogaster*, for example, males have been shown to produce and deposit 9-tricosene (9-C23:1) on valuable food sources, thereby causing females to aggregate and lay eggs there^12^. Besides aggregation, pheromones can also cause sexual attraction or repulsion^1,4,13^. In *D*. *melanogaster*, CHC profiles are strongly sexually dimorphic and only males produce 7-tricosene (7-C23:1), a non-volatile CHC, and *cis*-11-octadecenyl acetate (cVA), a (non-CHC) volatile sex pheromone. As both of these compounds are transferred to females during copulation^14–16^, other males take the presence of these compounds as cues for females having already mated, resulting in them acting as anti-aphrodisiacs and courtship-inhibiting pheromones for other males^17–20^. By contrast, some alkadienes, such as 7,11-dienes, are exclusively produced by female *D*. *melanogaster*. These compounds have an aphrodisiac effect and provoke courtship behavior in *D*. *melanogaster* males^20–22^. Finally, the different and complex CHC profiles have also been shown to play a role in species recognition in flies, moths, and various other insects^23–32^.

*Drosophila melanogaster* CHC profiles have been extensively studied over the last decades and numerous factors have been shown to impact CHC composition, including age, sex, maturation, interaction with the opposite sex, temperature, food, and hormones^14,33–37^. Of these, age, maturation, sex and social experience play a prominent role in determining the nature and the quantity of CHCs that are present. Aging, and in particular maturation with age, accounts for large differences in CHC profiles, and these have been shown to affect the sexual attractiveness of the flies^33^. Sex of the fly is a second important determinant of the CHC profile. As mentioned earlier, in *Drosophila melanogaster*, the CHC profile is strongly sexually dimorphic, with 7-C23:1 only being produced by males, and 7,11-dienes only by females. The (non-CHC) volatile sex pheromone, cVA, is also only produced by males^14,21^. However, other *Drosophila* species such as *Drosophila serrata*, *Drosophila takahashii* and *Drosophila pseudoobscura* show a monomorphic CHC profile^38^. In these species, both males and females produce the same CHCs, but not necessarily in the same abundance. Finally, a third important factor regulating CHC production is interaction with the opposite sex, which has been shown to impact the CHC profile either as a result of the direct transfer of CHCs between the sexes or indirectly by affecting CHC production, e.g. following mating^10,34,39^.

The current study focuses on the spotted wing *Drosophila*, *Drosophila suzukii* (Matsumura) (Insecta: Diptera: Drosophilidae), which is a pest species that originates in Southeast Asia and has been spreading throughout Europe and the US since 2008^40–42^. The females possess a serrated ovipositor, which enables them to cut through the skin of fruits such as cherries, blueberries and strawberries and deposit fertilized eggs inside the ripening fruits^43,44^. As yet, very little is known about the chemical communication and pheromones used by *Drosophila suzukii*. Previously, it has been reported that *Drosophila suzukii* has a monomorphic CHC profile and that cVA, the volatile anti-aphrodisiac pheromone produced by *Drosophila melanogaster* males, is not produced by *Drosophila suzukii*^45^. In a separate study, it was shown that total CHC content increases in *Drosophila suzukii* females with maturation and that this may be relevant for reproductive behavior^46^. Based on these studies, we hypothesized that ─ analogous to what is observed in other insect species^47^ ─ cuticular hydrocarbons (CHCs) could be acting as pheromones regulating sexual behavior in *Drosophila suzukii*. Hence, the aim of this study was to determine how ageing and maturation, sex and interaction with the opposite sex affect the CHC profiles of *Drosophila suzukii*. The experiments show that there are prominent changes in CHC profiles in function of age-related maturation and less prominent changes in function of sex or interactions with the opposite sex. Subsequently, the biological significance of the changes in CHC profiles was tested by applying (“perfuming”) distinct CHCs or blends of CHCs onto the cuticle of *Drosophila suzukii* and determining the impact on courtship behavior and copulation.

## Results

### Identity of *Drosophila suzukii* CHCs

**Figure 1:**
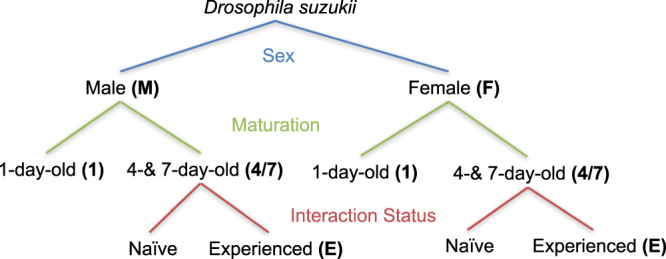
*Drosophila suzukii* sampling scheme used. The schematic shows the 10 different types of *D*. *suzukii* flies that were collected for GC-MS samples. These samples were collected to be able to analyze the effect of sex (blue), age and age-dependent maturation (green) and interaction status (red) on observed cuticular hydrocarbon profiles. The bold labels indicate the sample names used throughout the rest of this manuscript. To distinguish interaction naive from interaction experienced flies we added E for the experienced flies.

**Figure 2:**
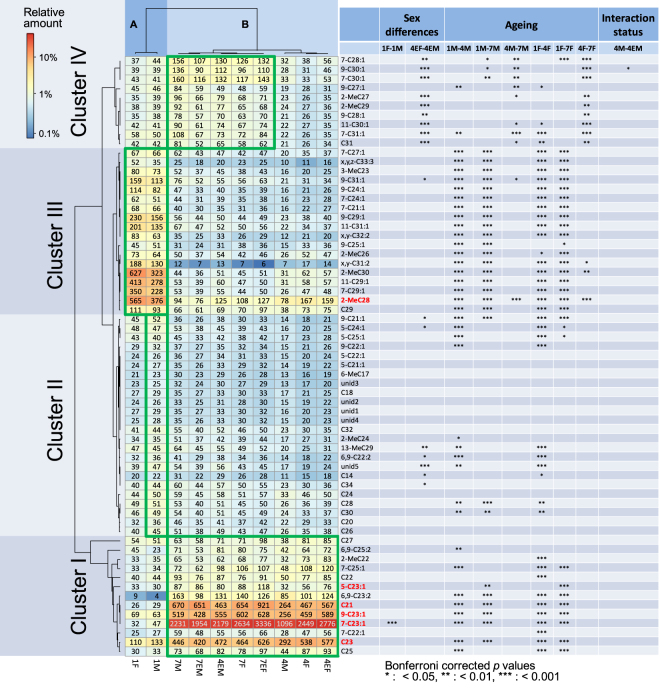
Cuticular hydrocarbon profiles of *D*. *suzukii* as a function of sex, age and interaction status. Relative quantities of different cuticular hydrocarbons produced by *D*. *suzukii* males (M) and females (F) of different ages (1, 4 or 7 day old) and interaction status (E = socially experienced). Clusterings use UPGMA hierarchical clustering and one minus the Pearson correlation as the distance metric. The heat map shading reflects the relative amount (in %) produced of each compound and the values are the geometric mean absolute amounts (in ng) produced per individual (n = 4 samples per group with 5 individuals pooled per sample; geometric means were used because of the strictly positive measurement scale). The significance of differences in relative abundance are shown for selected contrasts when the absolute fold difference was greater than 1.6. Relative compound abundances show strong age-related variation, but only weak differences related to sex or interaction status (based on per-compound robust linear models on log10 transformed relative abundances with Bonferroni *p* value correction and with class coded as a fixed factor; robust linear models as opposed to regular ANOVAs were used to allow for small amounts of heteroscedasticity observed for some compounds). Contrasts 4F-4M, 7F-7M, 7EF-7EM (sex differences) and 4F-4EF, 7M-7EM, 7F-7EF (social experience) were also calculated, but are not shown as these did not show any significant fold differences larger than 1.6. The colors represent the relative abundance of the compounds listed on the right-hand side of the table and the types of samples analyzed are shown at the bottom.

CHC profiles of *Drosophila suzukii* corresponding to ten conditions differing in sex (male and female), age (one, four- and seven-day-old) and interaction status (interaction or no interaction with the opposite sex) were analyzed using Gas Chromatography – Mass Spectrometry (GC-MS) (Fig. 1). In total, we quantified 65 chromatographic peaks, of which we were able to confidently identify 60 as linear or monomethyl-branched alkanes, monoenes, dienes or trienes (see Supplementary Table S1 for a complete list). In addition to the 25 that were previously identified^45^, we found an additional 35 that were not previously described for *Drosophila suzukii*. Heat maps of the relative amounts of each compound were generated to visualise the observed patterns (Fig. 2 and Supplementary Figure S1). Observed differences in relative abundance as a function of sex, age and interaction status, tested using robust linear models (*rlm*s) and posthoc tests with Bonferroni *p* value correction, are also given in Fig. 2 and will be discussed in more detail below.

### Limited effect of sex and interaction status on CHC profiles

From our data, we can conclude that *Drosophila suzukii* has a sexually monomorphic CHC profile in which none of the compounds show sex-specific qualitative differences, and males also do not produce cVA, consistent with^45^ (see columns “Sex differences” in Fig. 2). However, a few quantitative differences can be observed. For example, the comparison between one-day-old males and females (1 M vs. 1 F) shows a significant difference for 7-C23:1 (7-tricosene), the most abundant CHC in both males and females (Fig. 2). Differences between the sexes were found in the comparison of four-day-old interaction experienced females vs. males (4EF vs. 4EM). These differences mainly occur among C27 to C31 monoenes and methylated alkanes (Fig. 2). For the comparison of four-day-old or seven-day-old males vs. females (4M vs. 4F, 7M vs. 7F and 7-EF-7EM), no significant differences are observed.

To look at the effect of interaction status in terms of CHC production we compared four-day-old and seven-day-old flies that did or did not interact with the opposite sex (4F vs. 4EF, 7F vs. 7EF, 4M vs. 4EM and 7M vs. 7EM). Only in the comparison 4 M vs. 4EM did we find a significant difference for the compound 9-C30:1 (see column Interaction status in Fig. 2). However, in light of the results from the other social interaction comparisons, we conclude that interaction status (i.e. interaction with the other sex) does not alter the CHC profile composition in *Drosophila suzukii* in a major way.

### CHC differences linked to age-dependent sexual maturation

Contrary to sex and experience, age and age-dependent maturation have very strong effects on CHC production in both males and females (Fig. 2), especially when comparing sexually immature one-day old with sexually mature four- or seven-day old flies (see columns on “Ageing” in Fig. 2). These findings are consistent with the previously reported increase in total CHC with age^46^. Many significant differences were found when comparing one-day old females and males to mature four- and seven-day old flies (1M vs. 4M, 1M vs. 7M, 1F vs. 4F and 1F vs. 7F). Significant differences were mainly found in co-regulated compound Clusters I and III (Fig. 2). Cluster I contains the compounds that most strongly increase in relative abundance with age, whereas Cluster III contains mostly compounds that decrease in relative abundance with age (see color shading in the heat map of Fig. 2).

Comparisons between four- and seven-day-old flies show significant differences mainly in a set of longer-chain cuticular hydrocarbons with chain lengths ranging from C27 to C31, situated mostly in compound Cluster IV. Since the natural abundance of these compounds is lower, we reasoned that the compounds differing between one day old and four day old flies are more likely to exhibit potential pheromonal functions since this time window corresponds to sexual maturation.

Overall, we conclude that ageing and maturation are important determinants of the composition of the CHC profile. Furthermore, we hypothesize that the compounds that change significantly with maturation during the first four days may contribute to the chemical signaling of sexual maturity and potentially act as pheromones. To test this hypothesis, the compounds marked in Fig. 2 in bold red were selected for further behavioral testing.

### The role of CHCs in courtship and mating behavior

**Figure 3:**
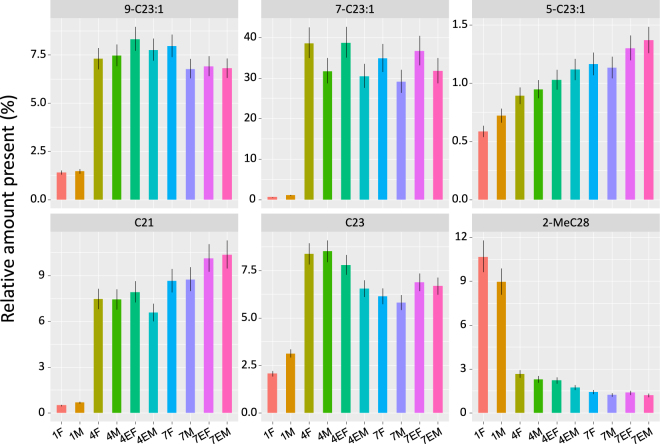
Differences in relative abundance of the six compounds tested in behavioral bioassays. Values shown are the least square mean relative abundances with 95% confidence intervals as calculated from the fitted robust linear models (calculated on a log10 scale and then backtransformed to the original scale).

**Figure 4:**
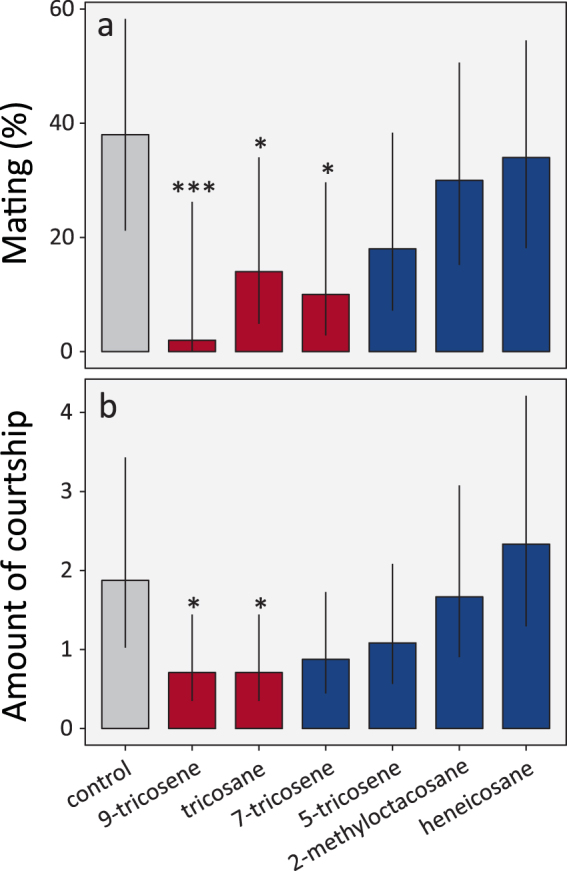
Disturbing C23 hydrocarbons in female *D*. *suzukii* results in loss of recognition during mating. Perfuming single four-day old *D*. *suzukii* females with 1312 ng (*SD* = 356 ng, *n = *5, ca. 10 µl of 100 ng/µl in hexane) of 9- and 7-tricosene and tricosane results in the partial loss of recognition during mating, as is shown by a reduced probability (**a**) to mate or (**b**) a reduced number of male courtship events, when placed together with four 4-day old males (estimated means and 95% confidence limits). In addition, treatment with 5-tricosene caused a trend to reduce the probability of mating and the amount of courtship observed, though this was not significant. By contrast, there was no evidence for two other prominent compounds, 2-methyloctacosane and heneicosane, to play any role in mate recognition. Significant differences with the hexane solvent control are based on Firth penalized-likelihood logistic regression models (**a**) (used to deal with the lack of variation in the 9-tricosene response, where none of the flies mated) and a negative binomial generalized linear model (**b**) (to take into account overdispersion) and are indicated with asterisks and are highlighted in red (*p* values: *** < 0.001, ** < 0.01, * < 0.05, n = 24 replicate trials per treatment).

Based on our analyses, we selected six compounds to be experimentally tested in behavioral bioassays, five of which showing a large increase in relative abundance as a function of age and maturation, namely 9-tricosene (9-C23:1), 7-tricosene (7-C23:1), 5-tricosene (5-C23:1), *n*-tricosane (*n*-C23) and heneicosane (C21), and one of which, 2-methyloctacosane (2Me-C28), showing a pronounced reduction with age (Figs 2 and 3). To test whether any of these six CHCs are actual pheromones that regulate courtship or mating, we used perfuming experiments, in which we applied a hexane solution of the pure compounds onto the abdomen of four-day-old naive females (4F). Females were perfumed to check for possible (anti-)aphrodisiac effects on male courtship and mating. These perfumed flies were paired with wild-type four-day-old interaction naive males, after which courtship and mating were scored over a period of two hours. A significant decrease in mating (expressed as the percentage that mated) compared to the hexane solvent control was found when the females were perfumed with 9-C23:1, C23 and 7-C23:1 (Firth’s penalized logistic regression, Fig. 4a). In addition, 9-C23 and C23 also significantly decreased the amount of courtship observed, measured as the sum of courtship bouts or individual courtship events recorded over the observation period of two hours (negative binomial GLM, Fig. 4b).

**Figure 5:**
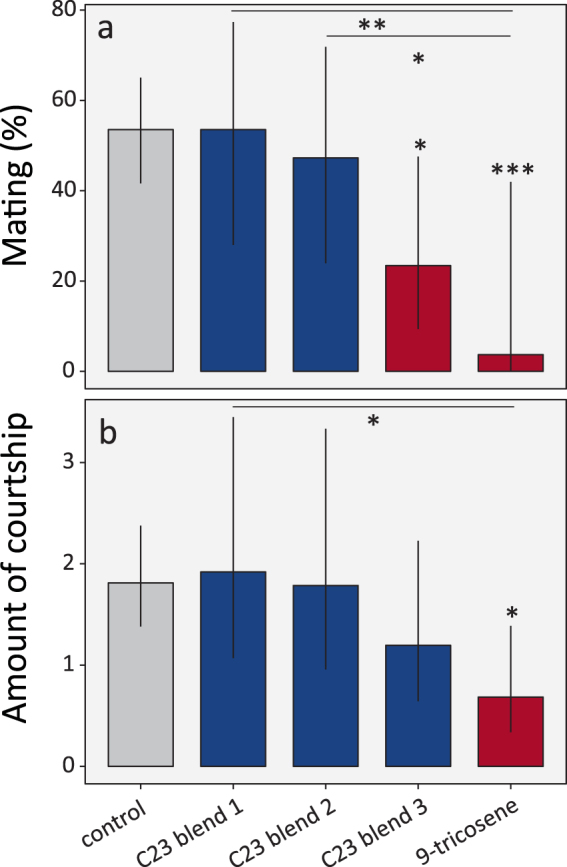
Perfuming *D*. *suzukii* females with C23 alkanes and alkenes (9-, 7- and 5-C23:1) in naturally occurring ratios preserves mate recognition. Perfuming single four-day old *D*. *suzukii* females with the natural ratio of C23, 5-C23:1, 7-C23:1 and 9-C23:1 (C23 blends 1, 2 and 3) does not alter mate recognition whereas C23 blend 3 (only for mating – panel a) and a 1312 ng (SD 356 ng (n = 5), 10 µl of 100 ng/µl in hexane) 9-tricosene treatment results in the partial loss of recognition during mating, as is shown by (**a**) a reduced probability to mate and (**b**) a reduced number of male courtship events observed, when placed together with four 4-day old males (estimated means and 95% confidence limits). Blends 1 to 3 all contained C23 alkanes and 9-, 7- and 5-C23:1 alkenes in naturally occurring ratios, but comparised different, increasing total doses of hydrocarbons: blend 1: 153 ng 9-C23:1, 865 ng 7-C23:1, 21 ng 5-C23:1 and 174 ng C23; blend 2: 459 ng 9-C23:1, 2449 ng 7-C23:1, 56 ng 5-C23:1 and 538 ng C23; blend 3: 1312 ng 9-C23:1, 6999 ng 7-C23:1, 160 ng 5-C23:1 and 1538 ng C23. Significant differences with the hexane solvent control are based on Firth penalized-likelihood logistic regression models (**a**) (used to deal with the lack of variation in the 9-tricosene response, where none of the flies mated) and a negative binomial generalized linear model (**b**) (used to take into account overdispersion) and are indicated with asterisks and are highlighted in red (*p* values: *** < 0.001, ** < 0.01, * < 0.05, n = 24 replicate trials per treatment).

Overall, these results show that the four tested CHCs with a chain length of 23 carbons (“C23-CHCs”) all negatively regulated courtship and mating behavior in *D*. *suzukii*. As an explanation of these results, we hypothesized that the application of individual C23-CHCs on the abdominal cuticle of the female resulted in the disruption of the mate recognition process by altering the naturally occurring ratios of the different C23 alkene ratios or the C23 alkane:alkene ratio. If this hypothesis is correct, we predicted that the addition of all C23-CHCs in their naturally occurring ratios should not affect mate recognition. To test this hypothesis, we perfumed flies with three distinct hexane solutions containing the C23-CHCs in their natural ratios (C23 blends 1 to 3). In C23 blend 1 the total amount of CHCs used was 1312ng, which was the same amount that was used in the single compound behavioral assay. C23 blend 2, by contrast, simply doubled the natural abundance of CHCs present on the four-day-old naive females (4 F) flies. Finally, C23 blend 3 contained the same amount of 9-tricosene used as in the single compound behavioral assays, i.e. 1312 ng, and the other C23-CHCs were administered in appropriate amounts to maintain the natural ratio (see legend of Fig. 5 for details). Hexane solvent perfumed females were used as a negative control and 9-C23:1 perfumed females were used as positive controls. Consistent with our hypothesis that the ratio of the C23-CHCs controls mate recognition in *D*. *suzukii*, we found that perfuming with C23 blends 1 and 2 did not significantly decrease mating compared to the solvent-only control (Firth’s penalized logistic regression, Fig. 5). Furthermore, both C23 blend 1 and 2 induced significantly more mating behavior than in the 9-tricosene positive control treatment (Fig. 5a). Furthermore, treatment with C23 blends 1, 2 or 3 did not significantly reduce the total amount of courtship observed compared to the solvent-only control, whereas treatment with C23 blend 1 resulted in significantly more courtship than observed following treatment with the 9-tricosene positive control (negative binomial GLM, Fig. 5b). The small but significant decrease in mating observed following treatment with C23 blend 3 is likely due to the extremely high total amount of CHCs added in that treatment, which deviates very much from the natural abundance. Based on these results, we conclude that the C23-CHCs ratios play a role in regulating *Drosophila suzukii* mating and courtship behavior, and that interfering in their naturally occurring ratios disrupts mate recognition. Total abundance of the CHCs, however, may also play a role, but this effect was less pronounced.

## Discussion

In the present study, we made a detailed characterization of the cuticular hydrocarbon profiles of male and female *Drosophila suzukii* as a function of age, sex and interaction with the opposite sex, and tested six pure compounds which strongly differed in function of maturation for their effects on courtship and mating. Overall, we found 35 CHCs never before identified in *Drosophila suzukii*, yielding a total of 60 CHCs present on the cuticle of *Drosophila suzukii*. Our data confirm that *Drosophila suzukii* has sexually monomorphic CHC profiles, with no identified compounds unique to males or females and no strong quantitative differences between males and females. We find high levels of monoenes, similar to previous observations in other monomorphic *Drosophila* species^48^ and we also confirm the absence of cVA^45^. A few compounds require further research to fully confirm their structure or the positions of the double bonds of the different isomers. Importantly, bioassays further showed that perfuming females with C23 linear alkanes and alkenes disrupts mating and courtship, and that this was likely due to the disruption of the natural ratios in which these compounds normally occur. Hence, these experiments showed that these CHCs play an important role in mate recognition in *D*. *suzukii*.

We focused on three very important variables affecting CHC profiles, namely age (and age-dependent maturation), sex and interaction with the opposite sex. Our most important observation was that ageing, and age-dependent maturation, appears to be the primary determining factor of differences in CHC production, whereas the sex of the fly and interaction with the opposite sex revealed few significant differences. Major changes in CHC profile were observed when comparing both one- and four-day old flies and four-day-old flies to seven-day old flies, and this both in males and females. Our observations are consistent with earlier work showing a higher abundance of overall CHCs in females upon maturation^46^.

The most abundant compounds in the *Drosophila suzukii* CHC profile were C23 alkanes and alkenes, which have been described to play a pheromonal role in other *Drosophila* species, including in *Drosophila melanogaster*, *Drosophila sechellia*, *Drosophila simulans* and *Drosophila lummei*^49–51^. Not only were these C23 alkanes and alkenes the most prominent compounds in the CHC profiles of *Drosophila suzukii* in every sample group, they also showed the largest significant increase with aging. Given their implication as pheromones in other *Drosophila* species, we hypothesized that these compounds could act as sex pheromones, fertility markers or play a role in mate recognition, and that disruption of C23 levels would therefore affect courtship and mating.

In our bioassays, we did indeed find a large effect for some of the compounds tested, with especially 9-C23:1 and to a lesser extent also 7-C23:1 and *n*-C23 strongly affecting mating behavior. Nevertheless, in contrast to our a priori hypothesis, mating and courtship were disrupted and not enhanced if females were perfumed with these compounds. At first glance, this would seem to imply an anti-aphrodisiac effect, similar to what has been observed for the male-specific 7-C23:1 in *Drosophila melanogaster*, which is transferred during copulation by the males, and inhibits courtship when present on females^19,52^. In our case, however, a true anti-aphrodisiac effect in *Drosophila suzukii* is highly unlikely given that (i) *Drosophila suzukii* females also produce this compound and (ii) that we found no evidence for actual transfer of these compounds from males to females.

As an alternative hypothesis, we proposed that the ratio among the different C23-CHCs is used as a cue in mate recognition, and that the inhibition of mating and courtship is the result of the disruption of the natural ratios caused by the addition of single compounds onto the cuticle of individual flies. In line with this hypothesis, we found that the addition of the C23-CHCs in their natural ratio and abundance (i.e. a doubling of their natural absolute abundance) did not affect courtship and mating behavior compared to the controls, in contrast to the strong inhibition of courtship and mating that is observed when flies were perfumed with individual C23-CHC compounds. From this experiment, we propose that the ratios of the C23-CHC compounds play an important role in *Drosophila suzukii* species and/or mate recognition^25,53,54^.

Since CHCs are short-acting non-volatile compounds, their role in chemical communication is limited to short range and is contact and gustation-dependent^4^. We cannot exclude the presence of other shorter volatile compounds being present that may act as pheromones. In *Drosophila melanogaster* it has recently been shown that short volatile compounds, such as methyl laurate, are not detected by conventional GC-MS methodology, but do play an important role in mating behavior^54^. Since the production of these short volatile odorants is conserved in different *Drosophila* species such as *Drosophila simulans*, *Drosophila erecta*, *Drosophila mauritiana* and *Drosophila yakuba*, a similar longer-range pheromone could also be present in *Drosophila suzukii*^55^.

Our findings offer potential new routes for integrated past management (IPM). Currently, basic apple-cider-vinegar traps are used for monitoring purposes^56^. However, given the rapid spread of *Drosophila suzukii* control methods are urgently needed^40,57^. For most pest insects, IPMs are based on disrupting or manipulating chemical communication^58,59^. Many potential IPM strategies such as attract-and-kill^60^, mass trapping^61^ and mating disruption^62^ rely on chemical communication used by insects. We propose that C23 CHCs could be part of an IPM for *Drosophila suzukii* to cause disruption of mating or even species recognition.

## Material and Methods

### *Drosophila suzukii* fly husbandry

A wild-caught *Drosophila suzukii* strain was obtained from PCFruit (Proefcentrum Fruitteelt VZW, Sint-Truiden, Limburg, Belgium). The flies were reared in quarantined facilities at room temperature (21–22 °C) on a newly adapted food recipe composed of 1 L of water, 7 g agar-agar, 100 g cornmeal, 60 g sucrose, 15 g fresh yeast, and cooked for 20 minutes. 0.75 g nipagin (dissolved in 4 ml ethanol) was added to the food prior to distribution in vials. All flies were collected within 12 hours after eclosion, after which they were used to generate the one-day-old samples or further aged to four- or seven-days-old. In addition to sex and age, we also distinguished groups that were allowed to interact with the other sex versus ones which were not. We will refer to these groups as interaction naive and interaction experienced. The interaction naive flies were aged separate from the other sex, while the interaction experienced flies were age mixed with the other sex.

### *Drosophila suzukii* selection for GC-MS sample preparation

Age-dependent and maturation-associated changes were determined by comparing three time-points. One-day-old flies are immature, and are thus unable to mate or produce offspring. Four days was selected as the second time-point based on preliminary behavioral experiments. In these experiments, we analyzed courtship and mating of flies that were 1, 2, 3 or 4 days old. We observed a complete absence of courtship and mating in one and two day old flies, whereas in three day old and certainly four day old flies we consistently observed courtship and mating. We therefore concluded that at four days old, *Drosophila suzukii* are fully mature and will readily mate when exposed to the other sex, and, therefore, that the CHC profiles would signal this maturity. In addition, we also selected seven day old flies to determine whether the CHC profiles showed additional changes with increased age. We analyzed the CHC profiles of these three different age cohorts in both males and females to identify any effect of sex. Finally, to determine whether interaction with the other sex would impact CHC profiles (e.g. by direct transfer of CHCs), we also analyzed males and females that were either not previously exposed to the other sex (interaction naive) or those that had been (interaction experienced).

### Gas chromatography – mass spectrometry (GC-MS) analysis

To determine how age-related maturation, sex and interaction with the opposite sex affected CHC profiles, we subjected all samples to GC-MS analysis. Each individual GC-MS sample consisted of an extract of 5 flies of the same age, sex and interaction status, which was obtained by placing the 5 flies in 100 µL n-hexane (HPLC grade) in 2 mL GC vials (Agilent Technologies), vortexing the sample for one minute, and after ten minutes transferring the hexane extract to new 2 mL GC vials containing 150-µL glass inserts. Ten different types of samples were prepared, which differed in age, sex or interaction status (Fig. 1). Four biological replicates of pools of 5 flies each were analyzed per condition. This proved superior to analyzing extracts of individual flies, since analysis of individual flies did not allow quantification of many of the minor CHC compounds. Samples were analyzed on a gas chromatograph (Thermo Fisher Scientific Trace 1300 series) coupled with a mass spectrometer (Thermo Fisher Scientific ISQ series MS). We used an injection volume of 1 μL and pulsed splitless injection with an inlet temperature of 320 °C, a split flow of 80 ml/min, a surge pressure of 170 kPa for 0.8 min and a purge flow of 5 ml/min. The initial temperature of 40 °C was held for 2 min, then increased to 120 °C at a rate of 20 °C min^−1^, to 200 °C at a rate of 10 °C min^−1^, to 250 °C at a rate of 7 °C min^−1^, and finally to 350 °C at a rate of 5 °C min^−1^ which was held for 4 minutes. Helium was used as a carrier gas at a constant flow rate of 0.9 mL min^−1^. We used an ion source and MS transfer line temperature of 300 °C, a mass acquisition range of 33 to 580 and a scan rate of 6.7 scans/s. The column used was a Restek RXi-5sil MS 20 m one with an internal diameter of 0.18 mm and a film thickness of 0.18 μm. Peaks in the total ion chromatogram were integrated using a custom R script (available from the authors on request). External linear alkane ladders containing n-heptane (C7) up to n-tetracontane (C24) injected at three different concentrations (0.1 μg/μl, 0.01 μg/μl and 0.001 μg/μl) were used to perform absolute quantification of all hydrocarbons as well as calculate spline-interpolated retention indices^63^. For quantification, we used the nearest eluting linear alkane for each compound to construct a calibration curve, using a linear regression on a log(concentration) vs. log(peak area) scale (in all cases there was excellent linearity on a log-log scale). Hydrocarbons could be readily identified based on their expected fragmentation patterns as well as their expected retention indices, as given in Pherobase, the NIST2014 retention index library and previous literature (e.g.^64,65^, correcting for predictable differences between DB-1 and DB-5 type columns). Alkene and alkadiene double bond positions were determined using DMDS derivatisation where possible^66^.

### Behavioral assays to test the effect of individual CHCs on courtship behavior

Five cuticular hydrocarbons that showed a strong positive association, namely *n*-heneicosane (*n*-C21), *n*-tricosane (*n*-C23), 5-tricosene (5-C23:1), 7-tricosene (7-C23:1) and 9-tricosene (9-C23:1), and one CHC that showed a negative association with maturation, namely 2-methyloctacosane (2Me-C28), were shortlisted to be tested in pherome bioassays, to determine their effect on courtship behaviour. *n*-heneicosane (98%), *n*-tricosane (98%), 7-tricosene (98%) and 9-tricosene (97%) were obtained from Thermo-Fischer and Sigma Aldrich. 5-tricosene (99%) and 2-methyloctacosane (95%) were synthesized by Ecosynth (Gent, Belgium) as a service. All compounds were dissolved in *n*-hexane (95%) and applied on the abdomen (“perfumed”) of four-day-old *Drosophila suzukii* females using paintbrushes (number 2). The females were perfumed with ca. 10 µl of a CHC-solution of 100 µg/µl in hexane. By analysing flies perfumed with this solution using GC/MS analysis, we verified that the net amount of CHC transferred using this treatment was on average 1312.36 ng (*SD* = 355.74 ng, *n* = 5, range 874.42–1863.67 ng). The flies were perfumed 24 hours prior to testing. Behavioral assays to test the effect of these CHCs on courtship behavior were performed in a room with controlled temperature (21 °C) at the same time of day (10h30 am). The perfumed females were combined pairwise with a wild-type four-day-old *Drosophila suzukii* male in a circular arena (2 cm diameter). Sony Handycams CX240 were used to film every pair for two hours. This long recording time was used to compensate for the low activity rate of the species^67^. The videos were analyzed and scored for courtship behavior (chasing, wing spreading and wing flickering) and mating (copulation) based on the courtship modules described in^67^ and^68^. Courtship was scored as the number of courtship modules observed within the two hours of video-taping. Mating was scored for every female in a binary way, i.e. as 1 or 0 if mating did or did not occur. For every treatment, 24 independent couples were tested (cf^67,68^.). The analysis of the different C23 blend assays was done in three different batches. The first batch consisted of control versus 9-tricosene, the second batch was control versus C23 blend 2 and the final batch comprised control and C23 blends 1 and 3. No batch differences were observed in statistical analyses. The control depicted in Fig. 5 is the least-square mean of all 3 control batches.

### Statistical analysis

A heatmap showing mean differences among the different groups of flies was produced using R 3.4.0’s *pheatmap* package (Fig. 2). Tests for differences in the (log10 transformed) relative abundance of the different cuticular hydrocarbons^69^ as a function of sex, age or interaction status were based on robust linear model (*rlm*) analysis using R’s *MASS* package and posthoc tests coupled with Bonferroni *p*-value correction to correct for multiple testing (Fig. 2). To guard against possible false positives, we combined the criterion of Bonferroni-corrected *p-*values having to be below a nominal family-wise alpha level of 0.05 with a 1.6 absolute fold difference cutoff, as is commonly done in the genomics literature, and which in our case was appropriate given that chemical differences would have to be large for any compound to be able to serve as a reliable pheromone signal. We should mention that this procedure was slightly on the conservative side, since the Bonferroni correction assumes all tests were independent, whereas some dependency may exist due to some compounds being produced as a result of shared biosynthetic pathways. Robust linear models as opposed to regular ANOVAs were used to allow for small amounts of heteroscedasticity that were inferred to be present for some (14 out of 65) of the compounds based on Levene’s tests, and which robust linear models can effectively deal with via robust M-estimation^70^. However, a spread-level plot of the log of the absolute studentized residuals plotted against the log of the fitted values across all fits for all 65 compounds (*n* = 2600) showed that overall, the applied log10 transformation adequately stabilized the mean/variance relationship, and that a as a whole, there was no systematic relationship between the mean and the variance (see Supplemental Figure S2). In addition, generalized least squares analyses in which the mean-variance relationship was fitted from the data were less parsimonious based on the mean Akaike Information Criterion (−53 for *gls* vs. −102 for *rlm*). Furthermore, a Shapiro-Wilk’s W test for normality performed on the pooled studentized residuals of all 65 fits showed that overall, residuals were near-normally distributed (Shapiro-Wilk’s *W* = 0.97, which was ≫0.9, *n* = 2600). Overall, this meant that all underlying assumptions of the performed tests were met. The robustness of our analysis can also be seen from the fact that the difference in conclusion based on standard ANOVAs vs. those based on robust linear models was negligible, as 180 of the calculated contrasts with >1.6 fold differences were significant after Bonferroni *p*-value correction both in ANOVA analyses and in robust linear model analyses (Fig. 2), and that a robust linear model only returned a further 18 significant differences that were not significant based on standard ANOVA analysis.

The proportion of our pairs of flies in the perfumed vs. control condition where mating was observed were compared using bias-reduced Firth penalized-likelihood logistic regression models, as implemented in the *logistf* R package^71,72^. This was used instead of regular logistic regression to be able to deal with the lack of variation in the 9-tricosene treatment response, where none of the flies mated. No significant overdispersion was present in these analyses (see supplemental R script and data on Dryad: doi:10.5061/dryad.fc0m627). Finally, the frequencies of observing courtship in the perfumed vs. control conditions were compared using negative binomial GLMs using MASS’s *glm*.*nb* function. This was used to take into account a slight amount of overdispersion that was found in these analyses. For the trials where the different C23 blends were tested, experimental batch was included as an additional fixed factor.

## Electronic supplementary material

Supplementary information
