# Supplementary material for: The role of cuticular hydrocarbons in mate recognition in Drosophila suzukii
Source: Sci Rep. 2018 Mar 22;8:4996. doi: 10.1038/s41598-018-23189-6 (PMC5864920; doi:10.1038/s41598-018-23189-6)
Supplement: Supplementary file 1 — Supplementary information [file 41598_2018_23189_MOESM1_ESM.docx]

**The role of cuticular hydrocarbons in mate recognition in *Drosophila suzukii***

Yannick Snellings^1^, Beatriz Herrera^2^, Bruna Wildemann^1^, Melissa Beelen^1^, Liesbeth Zwarts^1^, Tom Wenseleers^3,*^, Patrick Callaerts^1,*^

^1^ KU Leuven, Department of Human Genetics, Laboratory of Behavioral and Developmental Genetics, Leuven, 3000, Belgium

^2^ VIB, Department of Microbiology, Laboratory of Systems Biology, Heverlee, 3001, Belgium

^3^ KU Leuven, Department of Biology, Ecology, Evolution and Biodiversity Conservation Section, Leuven, 3000, Belgium

*tom.wenseleers@kuleuven.be

*patrick.callaerts@kuleuven.be

**Supplementary materials**

**Supplementary table S1**

| **RI** | **Compound** | **Abbreviation** |
| --- | --- | --- |
| 1400 | n-tetradecane | C14 |
| 1712 | unidentified CHC1 | unid1 |
| 1755 | 6-methylheptadecane | 6-MeC17 |
| 1799 | n-octadecane | C18 |
| 1927 | unidentified CHC2 | unid2 |
| 1969 | unidentified CHC3 | unid3 |
| 1998 | n-eicosane | C20 |
| 2074 | 9-heneicosene | 9-C21:1 |
| 2080 | 7-heneicosene | 7-C21:1 |
| 2087 | 5-heneicosene | 5-C21:1 |
| 2098 | n-heneicosane | C21 |
| 2141 | unidentified CHC4 | unid4 |
| 2161 | unidentified CHC5 | unid5 |
| 2165 | 6,9-docosadiene | 6,9-C22:2 |
| 2174 | 9-docosene | 9-C22:1 |
| 2180 | 7-docosene | 7-C22:1 |
| 2197 | 5-docosene | 5-C22:1 |
| 2198 | n-docosane | C22 |
| 2261 | 2-methyldocosane | 2-MeC22 |
| 2278 | 6,9-tricosadiene | 6,9-C23:2 |
| 2279 | 9-tricosene | 9-C23:1 |
| 2282 | 7-tricosene | 7-C23:1 |
| 2293 | 5-tricosene | 5-C23:1 |
| 2298 | n-tricosane | C23 |
| 2372 | 3-methyltricosane | 3-MeC23 |
| 2379 | 9-tetracosene | 9-C24:1 |
| 2381 | 7-tetracosene | 7-C24:1 |
| 2385 | 5-tetracosene | 5-C24:1 |
| 2398 | n-tetracosane | C24 |
| 2461 | 2-methyltetracosane | 2-MeC24 |
| 2471 | 6,9-pentacosadiene | 6,9-C25:2 |
| 2473 | 9-pentacosene | 9-C25:1 |
| 2482 | 7-pentacosene | 7-C25:1 |
| 2492 | 5-pentacosene | 5-C25:1 |
| 2498 | n-pentacosane | C25 |
| 2598 | n-hexacosane | C26 |
| 2661 | 2-methylhexacosane | 2-MeC26 |
| 2676 | 9-heptacosene | 9-C27:1 |
| 2684 | 7-heptacosene | 7-C27:1 |
| 2697 | n-heptacosane | C27 |
| 2761 | 2-methylheptacosane | 2-MeC27 |
| 2780 | 9-octacosene | 9-C28:1 |
| 2785 | 7-octacosene | 7-C28:1 |
| 2798 | n-octacosane | C28 |
| 2861 | 2-methyloctacosane | 2-MeC28 |
| 2872 | 11-nonacosene | 11-C29:1 |
| 2877 | 9-nonacosene | 9-C29:1 |
| 2885 | 7-nonacosene | 7-C29:1 |
| 2897 | n-nonacosane | C29 |
| 2929 | 13-methylnonacosane | 13-MeC29 |
| 2960 | 2-methylnonacosane | 2-MeC29 |
| 2972 | 11-triacontene | 11-C30:1 |
| 2977 | 9-triacontene | 9-C30:1 |
| 2984 | 7-triacontene | 7-C30:1 |
| 2997 | n-triacontane | C30 |
| 3050 | x,y-hentriacontadiene (5 isomers) | x,y-C31:2 |
| 3061 | 2-methyltriacontane | 2-MeC30 |
| 3069 | 11-hentriacontene | 11-C31:1 |
| 3078 | 9-hentriacontene | 9-C31:1 |
| 3084 | 7-hentriacontene | 7-C31:1 |
| 3097 | n-hentriacontane | C31 |
| 3197 | n-dotriacontane | C32 |
| 3252 | x,y,z-tritriacontatriene (several isomers) | x,y,z-C33:3 |
| 3258 | x,y-tritriacontadiene (several isomers) | x,y-C32:2 |
| 3397 | n-tetratricontane | C34 |

**Supplementary Table S1: CHCs identified in *Drosophila suzukii* samples.** All 65 identified compounds plus their abbreviation and measured retention indices (RI) are shown.

**Supplementary Figure S1**


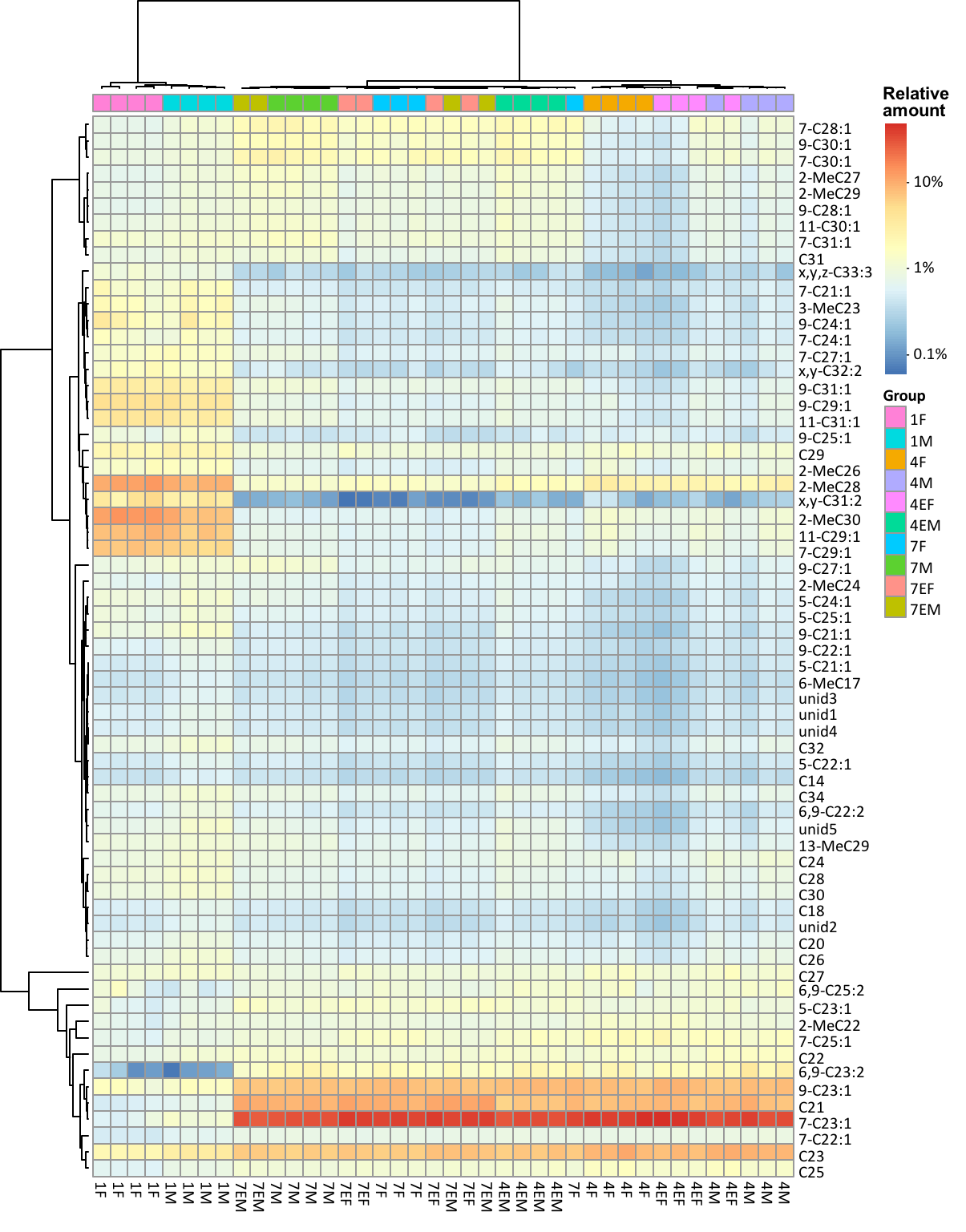


**Supplementary Figure S1: Cuticular hydrocarbon profiles of *D. suzukii* in function of sex, age and interaction status.** Heat map of the relative amounts of different cuticular hydrocarbons produced by *D. suzukii* males (M) and females (F) of different ages (1, 4 or 7 day old) and mating status (E= interaction experienced) as measured for all 4 replicate samples, each based on an extract of a pool of 5 individuals. Clusters use UPGMA hierarchical clustering and one minus the Pearson correlation as the distance metric. The heat map shading shows the relative amount (in %) produced of each compound.

**Supplementary Figure S2**

**Supplementary Figure S2: A spread-level plot based on the pooled studentized residuals obtained from the statistical analyses of all 65 compounds.** A spread-level plot in which the logarithm of the absolute studentized residuals, obtained from the robust linear models fitted on all 65 compounds, are plotted in function of the logarithm of the absolute fitted values reveals a nearly flat relationship. This demonstrates that there was no strong systematic relationship between the mean and the variance in our analysis.
